# Microstructure and properties of deposited metal for SUPER304H steel

**DOI:** 10.1038/s41598-017-14349-1

**Published:** 2017-10-31

**Authors:** QI. Yanchang, WU. Zhiquan, Zhang Xin, MA. Chengyong

**Affiliations:** 1 Welding Research Institute, General Iron and Steel Research Institute, Beijing, 100044 China; 2 Anhui Branch, China Datang Coporation, Anhui Hefei, 230088 China; 3 Institute of Thermal Power Generation Technology, China Datang Coporation Science and Technology Research Institute, Beijing, 102206 China

## Abstract

The microstructure and properties of Super304H weld joints were developed and studied. The Creq and Nieq composition points of the deposited metal were all located in the austenite region. The quantity and size of the Nb-phase in the deposited metal with 0.28wt.% Nb were lower than tnat in the other two deposited metals. The phases were mainly γ and Nb(C, N). The content of Nb(C, N) phases in the deposited metal with the 0.6 wt.% Nb content was approximately 2.2 times of the deposited metal with the 0.28 wt.% Nb content. The Nb-phases in the deposited metal with 0.28 wt.% Nb content were lower in quantity, weaker in the strengthening effect, of lower yield strength and lower in size than that the deposited metal with the 0.6 wt.% Nb content.

## Introduction

In order to improve the power efficiency of power plants, reducing CO_2_ emissions and consuming a hig amount of fuel, the turbine inlet steam parameters of supercritical and ultra-supercritical units with higher temperature (600 °C) and pressure (exceeding 31MPa)^1,2^ were developed. In contrast, the development of materials with long service life under high temperature and high pressure has become the key to the development of supercritical and ultra-supercritical units. With the constant construction of high parameter and high-sized capacity units, the new heat resistant steel has been widely utilized in the new unit. At present, a wide range of new heat resistant steels, mainly the ferritic heat resistant steels, such as the T91, P91, T92, P92, T122 and P122, as well as the austenitic heat resistant steel, such as the Super304H, TP347HFG and HR3C exist. In addition, the Cr, W, Cb and other alloying elements are added to the new heat resistant steel, whereas the high temperature creep rupture strength of the material is improved, which is the martensitic heat-resistant steel V^3–7^. The Super304H was developed by Sumitomo Steel Corp, which could meet the requirements of high temperature strength, oxidation resistance and long service life of the ultra supercritical parameters plant. The Super304H is suitable for superheaterd and reheater tubed and also is an economical austenitic stainless steel.

A high number of scholars believe^8,9^ that the Ni-based welding wire is the preferred welding material for the Super304H steel. Domestically and abroad for the welding of Super304H steels, the utilization of nickel based welding materials mainly includes the Inconel82 (ERNiCr-3), the Inconel625 (ERNiCrMo-3) and the Thermanit-617 (ERNiCrCoMo-3)^10^. The data indicate that^11^ the ERNiCrMo-3 and the ERNiCrCoMo-1 both contain molybdenum 8–10 wt.%. The molybdenum can improve the creep rupture strength of the weld metal, whereas simultaneously it will increase the precipitation tendency of the σ phase. The content of nickel in the Nickel based weld material highly exceeds the nickel content in the Super304H steel. The high nickel content of the weld metal can effectively control the σ phase precipitation, whereas the high nickel content will increase the production cost. In addition, the carbon content of three nickel based alloys was generally controlled at approximately 0.1 wt.%, whereas the impurity elements of S and P were controlled at 0.015 wt.% and 0.03 wt.% respectively.

The matching welding materials (YT-304H) of Super304H steel were developed by Japanese Sumitomo company. In order to improve the performance of the welding process, especially the weld metal crack resistance and the high temperature performance to be matched with the base metal, the compositions of YT-304H made the major adjustments: content improvement of manganese and nickel and addition of approximately 1 wt.% Mo^12,13^. The China Electric Power Research Institute^14^ developed the Super304H tungsten electrode argon arc welding welding wire, whereas the composition range of the welding wire and the mechanical properties at room temperature and the short-time tensile properties at 650 °C of the deposited metal are presented. The Super304H steel contains higher amount of alloying elements, which leads in the liquid metal viscosity increase. The mobility becomes worse, which contistutes the welding operation difficult. Due to the contents of Cr and Ni, the Super304H displays hot cracking tendency during welding. The melting point of the oxide film existing on the surface of the Super304H steel is higher than the Super304H steel melting point. Consequently, the film is not easy to melt in the liquid filled metal, easily forming slag or incomplete fusion during cool down^15^.

In the present work, the welding material which could match the SUPER304H heat resistant steel development, the effect of the proposed welding materials on the microstructure and properties of the weld for the SUPER304H steel were studied. In this paper, the microstructure and properties of three types of deposited metals design was studied.

## Material and Methods

### Experimental materials

Three solid welding wires were designed through smelting (Φ1.6), which were labeled as 1#, 2#, 3#. The SUPER304H austenitic heat-resistant steel was utilized in the test and the test board size was 430 mm × 200 mm × 20 mm. The test wires were utilized to deposite the cladding isolation layer of 8 mm in thickness on the groove and the pad surfaces, whereas the root clearance was 16 mm, which ensured that the deposited metal component would not be affected by the dilution of the substrate.

The chemical composition of the deposited metal was analyzed by ICP-AES and the results are presented in Table 1. The main contents of the three deposited metals differed in the contents of niobium, carbon and copper. In addition, the content of chromium and nickel in the 3# deposited metal was slightly lower than the contents of the other two types of deposited metal. Compared to the welding wire, the elements such as carbon, chromium and nickel were slightly lost; the nitrogen content was not increased whereas it decreased, which might be related to the nitrogen in the molten pool. The total amount of silicon and manganese was not changed, which indicated that the protective effect of the gas during the welding was good.

**Table 1:** chemical composition of welding wires (wt.%).

No.	C	Si + Mn	Cr	Ni	Mo	N	Nb	Cu
1#	0.10	3.54	17.77	16.10	≤1.2	0.13	0.61	2.95
2#	0.099	3.54	17.84	16.27	≤1.2	0.12	0.60	3.46
3#	0.080	3.39	16.97	15.24	≤1.2	0.13	0.28	2.91

### Experimental method

#### Test of welding wire deposited metal

**Figure 1:**
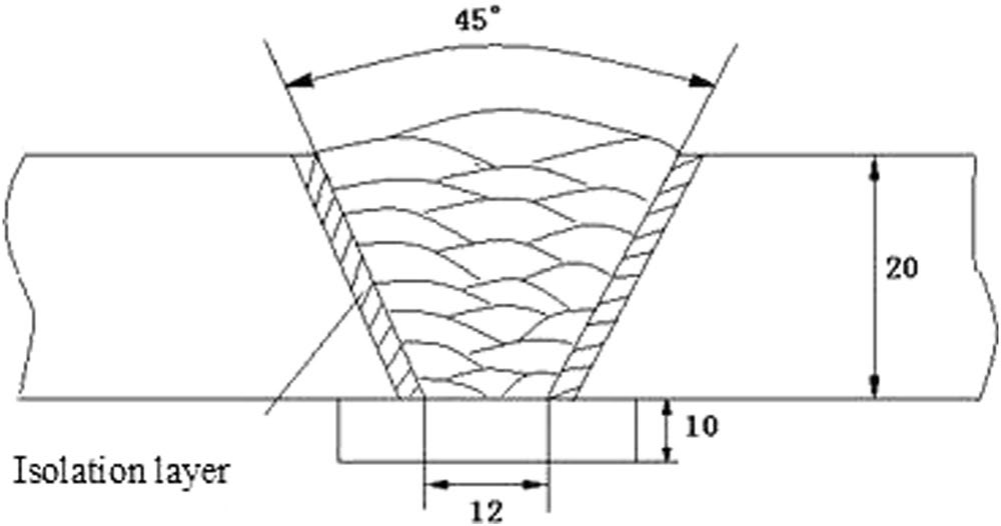
Schematic diagram of multi-layer welding.

The V belt plate groove was adopted as the welding plate to weld the deposited metal. The test plate material was T92 steel plate, and its size was 250 × 150 × 20 mm. The schematic diagram of V groove was shown in Fig. 1. In order to prevent the effect of the dilution of base metal on the component of welding wire deposited metal in the gas protection welding, the same testing welding wires was used for the isolation layer on both sides with surfacing thickness about 8 mm and for the TIG backing weld at bottom of the groove.

The AMET Maipulator welding machine with TIG automatic welding system was used to perform the pulsed TIG welding on the test plate. The shielding gas was 100% argon, and the gas flow was 15 L/min. Table 2 showed the welding parameters. Each welding joint set up 13 welding layers and 30 welding beads. The first 5 beads were the backing welds. The interpass temperature and wire feed rate were modified to keep the interpass temperature range 150–200 °C.

**Table 2:** welding process parameters of welding wire deposited metal.

Electric current/A	Voltage/V	Actual welding speed cm/min	Wire feeding speed mm/min	shielding gas	Gas flow L/min	Inter channel temperature °C
260	12.7	14	800	Pure Ar	15	≤100

#### Mechanical properties of deposited metal

**Figure 2:**
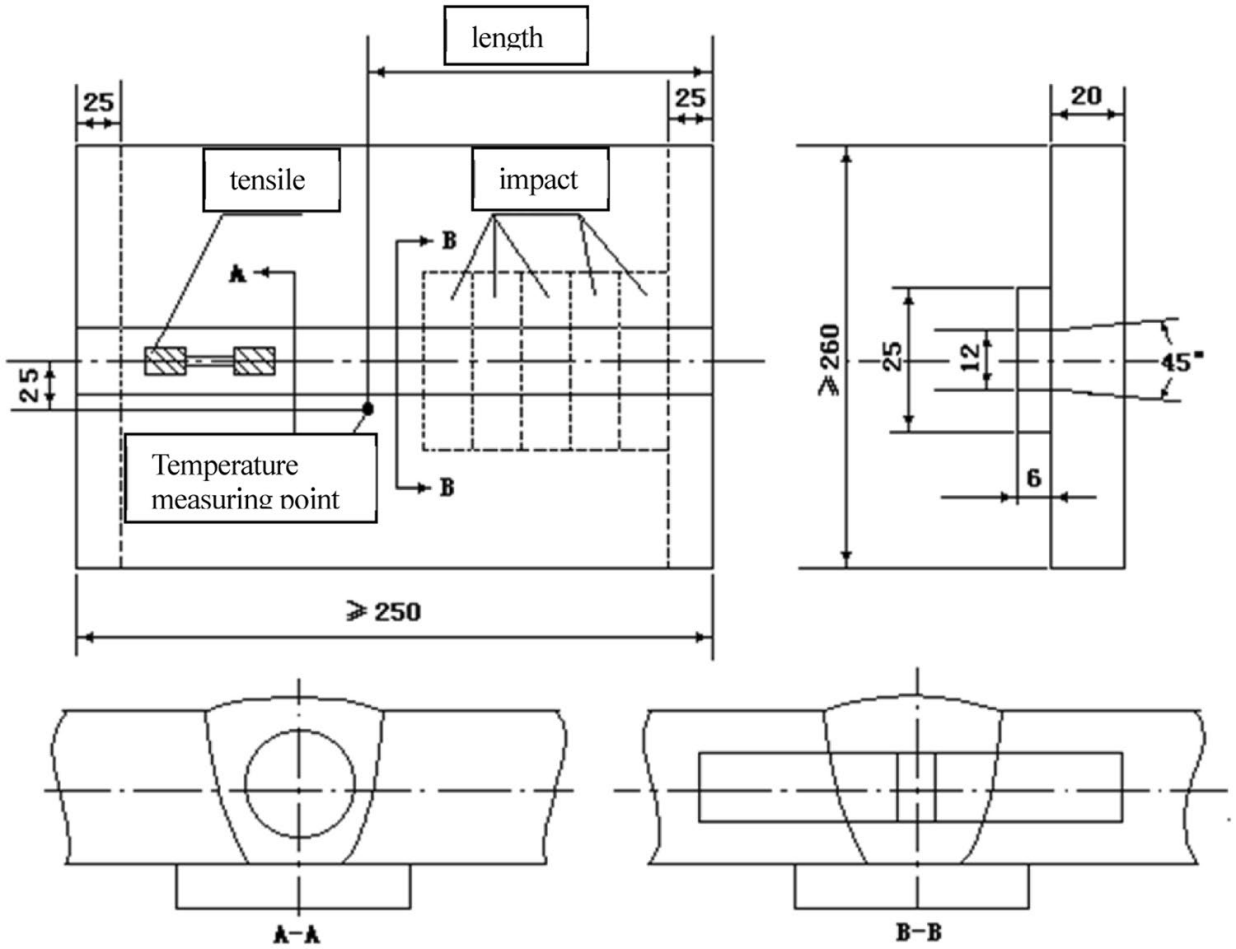
Location of deposited metal impact and tensile specimens.

Charpy shaped notch (V Notch V) test was used to test the impact toughness of the material by a single pendulum impact tests, and the performance index was the impact absorbing energy. From the tensile test, the mechanical properties could be obtained, including yield strength (R_P0.2_), tensile strength (R_m_), elongation after fracture (A%) and reduction of area (Z%). These indicators of mechanical properties were important for judging the toughness of material. Figure 2 indicated the location of deposited metal impact and tensile specimens. The impact tests were performed on JBN-300 test machine at high temperature (740 °C, 760 °C and 800 °C).

**Figure 3:**
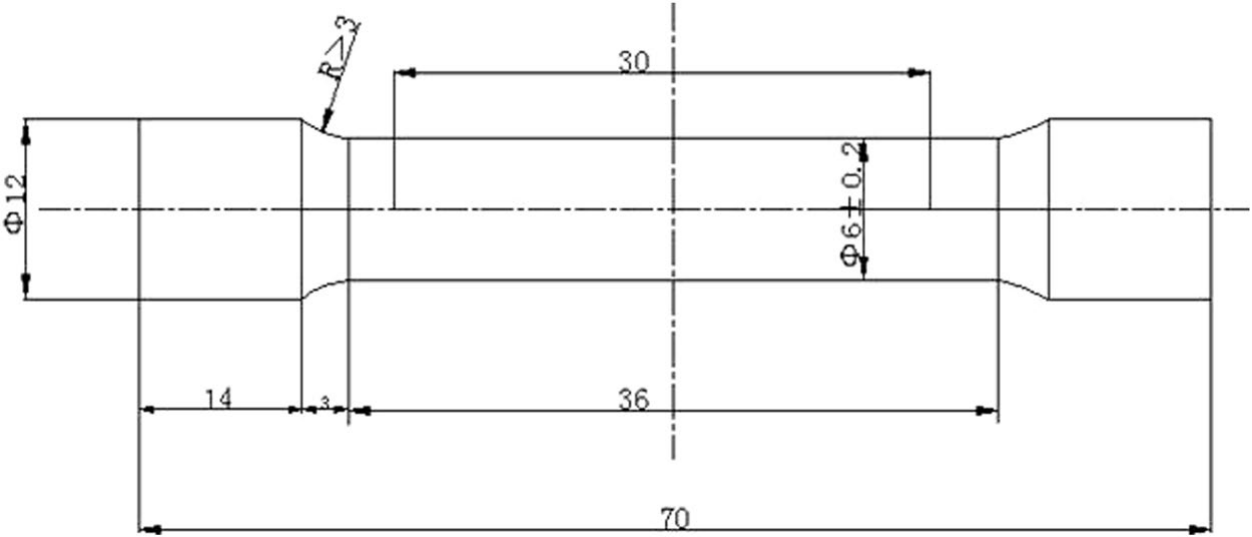
Sketch map of tensile samples processing.

Figure 3 gave the sketch map of tensile samples. The tensile specimens were manufactures as round bar specimens with a standard size of Φ6 × 105 mm. The tensile test was performed on UH-F50A (250kN) test machine at high temperature (740 °C, 760 °C and 800 °C).

#### Investigation on the microstructure and fracture of deposited metal

Spark emission spectroscopic method was adopted to analyze the alloy composition of deposited metal. The specimen for metallographic observation was cut along the longitudinal direction of welding pass. After grinding and polishing, the specimen was etched by the mixed solution consists of 5g CuCl_2_, 30 ml HCI, 25 ml alcohol and 30 ml H_2_O. Then, the microstructure of deposited metal was investigated by using the MEF-4M metallographic microscope and SCIAS 6.0 image analysis system. The second phase of deposited metal and impact fracture surface were analyzed using Scanning Electron Microscope of HITACHI S-4300 with matching Energy Dispersive Spectrometer.

In order to confirm the possible precipitated phase, the method of electrochemical extraction was used to study the second phase of deposited metal. In addition, PHLIPS APD-10X diffraction was also adopted for the analysis of phase composition of deposited metal.

#### Thermodynamics calculation on phase transition point of deposited metal and precipitates

Based on Thermo-calc software, the phase transition point of deposited metal and precipitates was analyzed considering the database of TCFE6. It should be noted that, the austenitizing temperature A_1_ under ideal condition was different from A_C1_ in Thermo-calc analysis, and A_C1_ which was under heat treatment condition was close to A_1_. Then, the analysis results were compared with the experimental results.

## Microstructure of deposited metals

### Solidification sub-grain boundary and Nb phase

**Figure 4:**
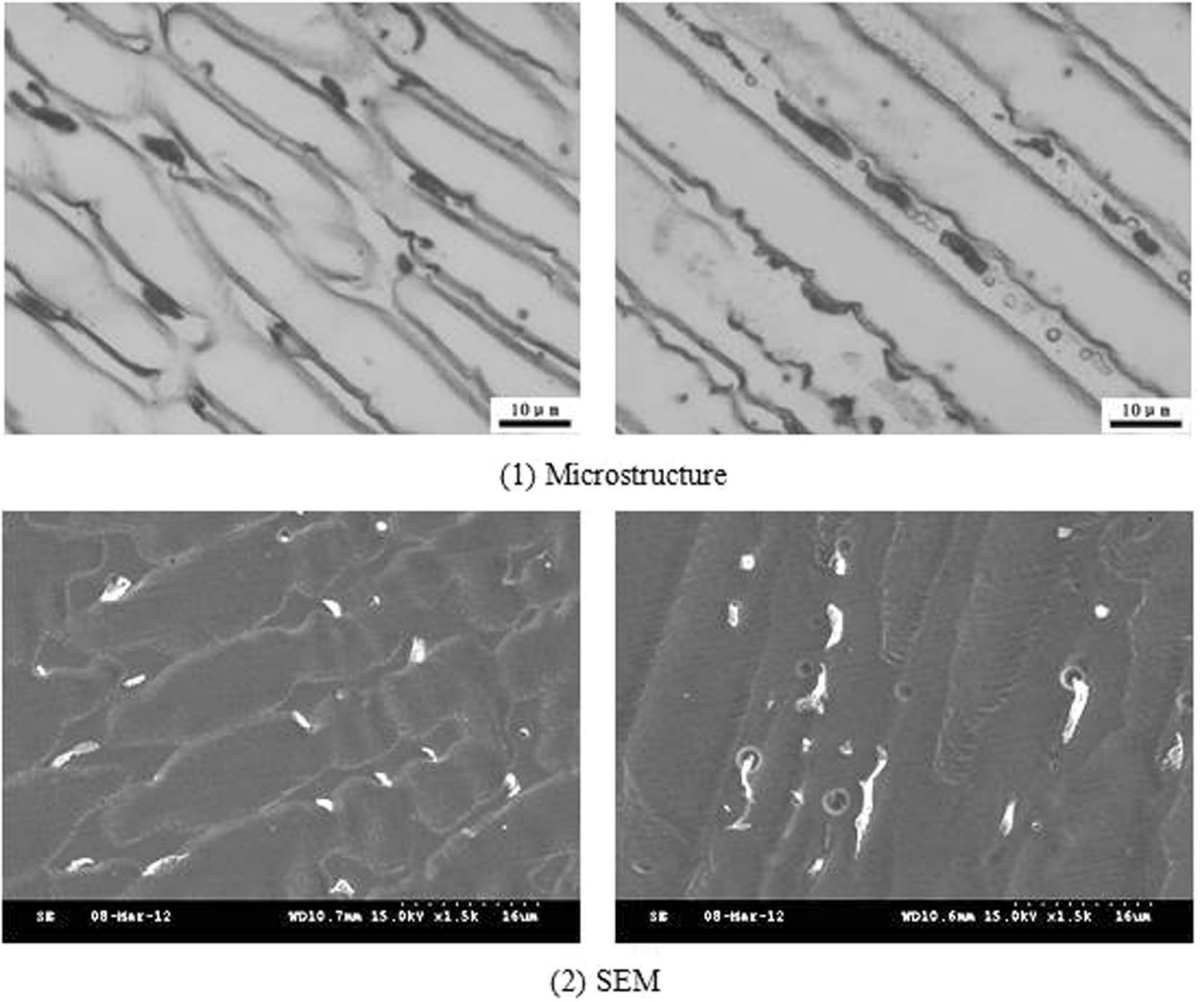
Different cross sections and Nb phases of sub-grain boundary in 3# deposited metal.

The solidification of the 3# deposited metal followed the austenite solidification mode and the crystal grains and cell shapes were clearly visible. Due to the segregation of alloying elements and impurities during solidification, the the segregation profile during solidification was preserved, as presented in Fig. 4. The Creq and Nieq of the deposited metals were 18 and 21.4, respectively. The set points were located in the phase diagram of the entire austenite region, which meant that the solidified deposited metal was austenite, whereas the microstructure was consistent with the metallographic structure. In Fig. 1, the upper convex part of the SEM morphology was the solidified subgrain boundary and the lower part is austenite. The micro-zone composition analysis was performed on the solidified sub grain boundaries and austenite grains, whereas the statistical results (mean values) are presented in Table 3. It was apparent that the content of the elements in the grain boundary was higher than the element contents in the austenite crystal and the distribution of the elements on the sub grain boundary obeyed the Scheil segregation. Due to the higher contents of chromium, nickel and copper on the sub boundary, the corrosion resistance was good, demonstrating a convex appearance subsequently to corrosion.

**Table 3:** Chemical composition of sub-grain boundary and austenite grain (wt.%)

Location	C	Si	Mn	Cr	Ni	Mo	N	Nb	Cu
sub-grain boundary	0.031	0.504	3.759	18.163	16.373	1.067	0.168	0.813	4.091
Austenite crystal	0.025	0.345	2.796	16.947	15.418	0.716	0.143	0.381	3.096

**Figure 5:**
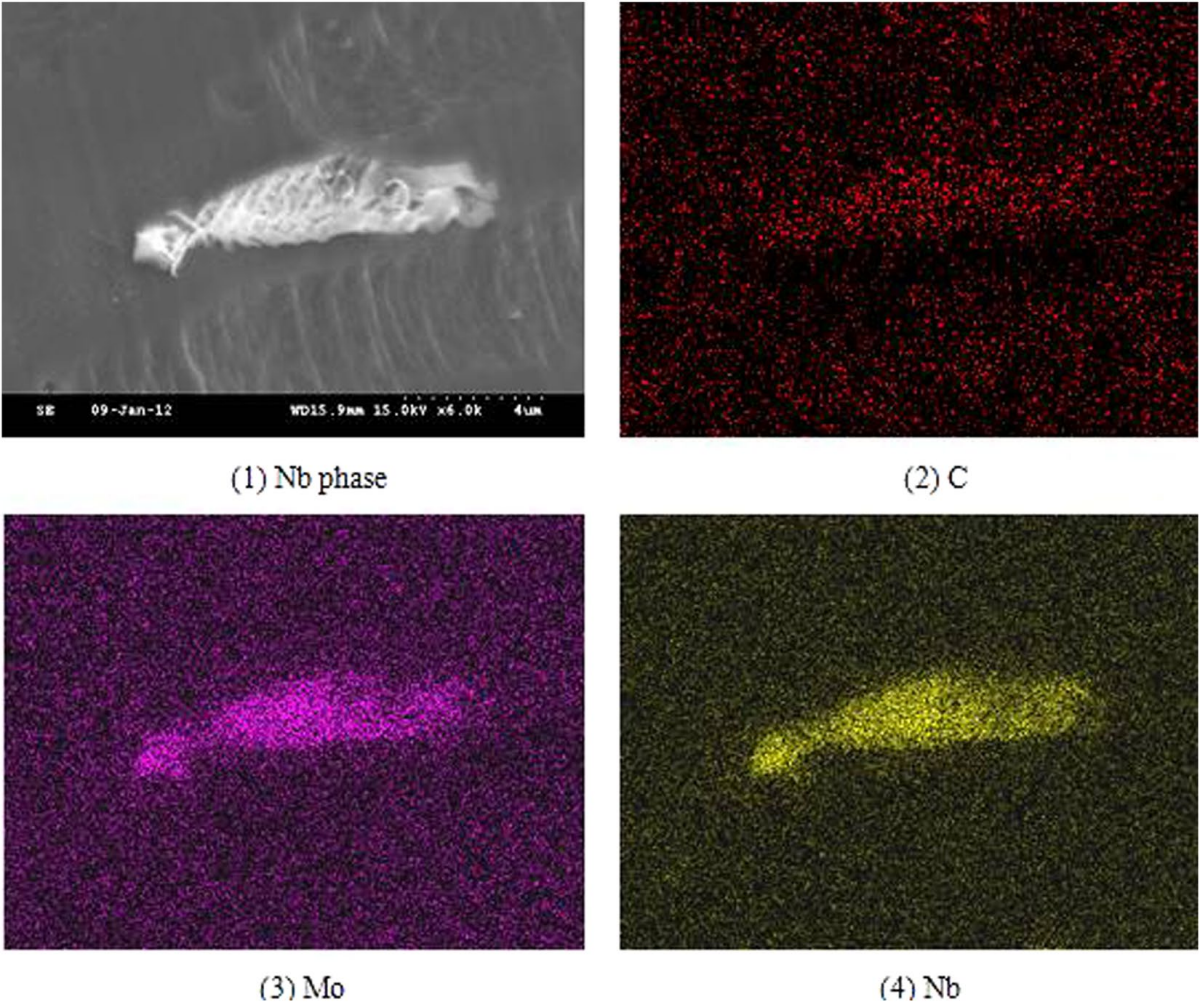
Elements distribution of niobium phase in 3# deposited metal.

A high number of precipitates were distributed on the solidified sub grain boundaries. The size of the precipitates exceeded 10 μm. The precipitated phase surface was not smooth and displayed a long or blocky shape. The element distribution of the precipitates was analyzed through the surface scanning, as presented in Fig. 5. The element distribution of the precipitated phase was analyzed through surface scanning, as presented in Fig. 5. It could be observed that the carbon and niobium elements had apparent segregation, which could be determined to be a niobium phase. Simultaneously, the segregation of molybdenum on the precipitated phase was observed and the content was approximately 2.8 wt.%. In addition, the distribution of nitrogen was also analyzed through surface scanning, whereas the signal was weak and no segregation was observed.

**Figure 6:**
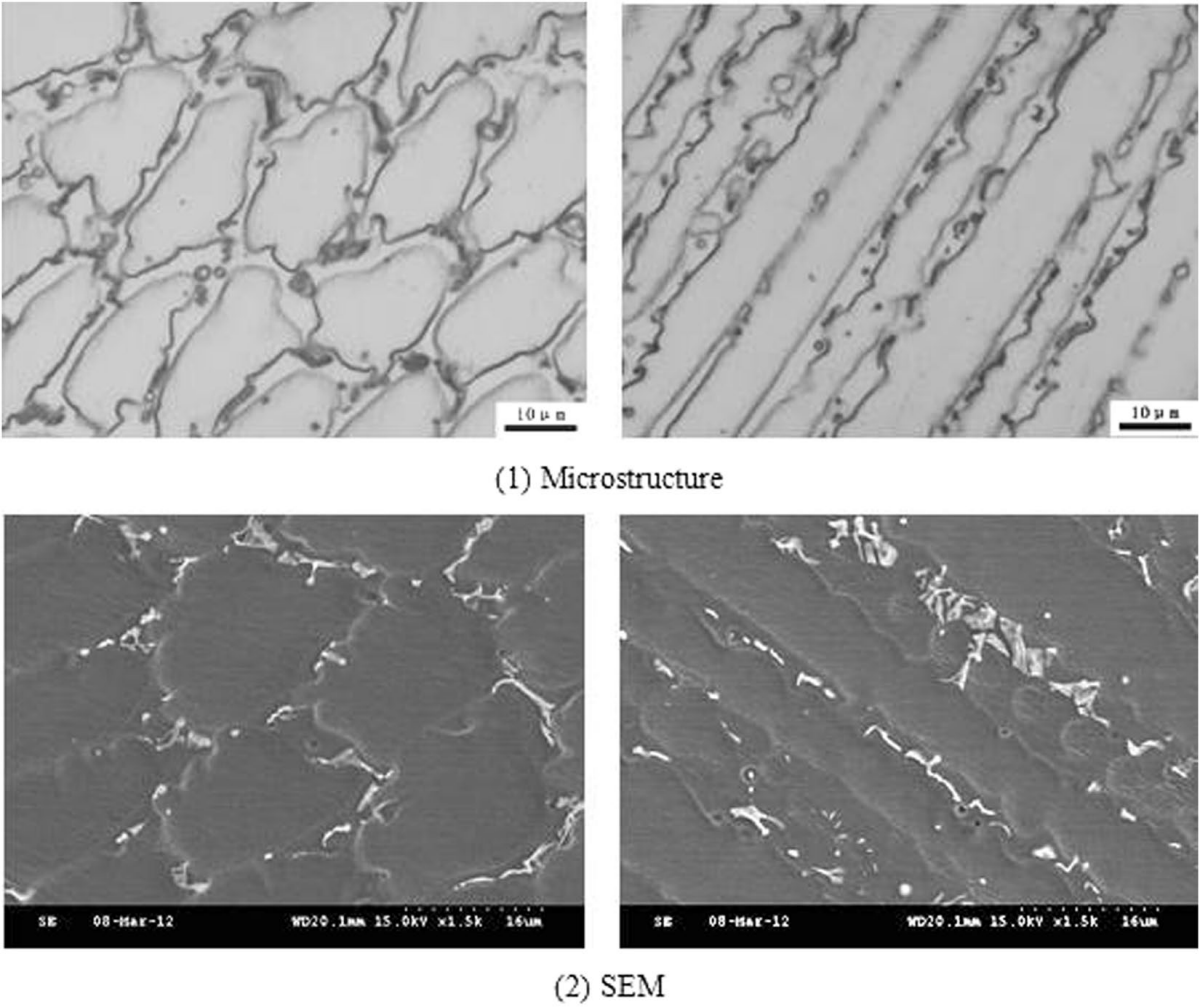
Different cross sections of sub-grain boundary in 1# deposited metal.

**Figure 7:**
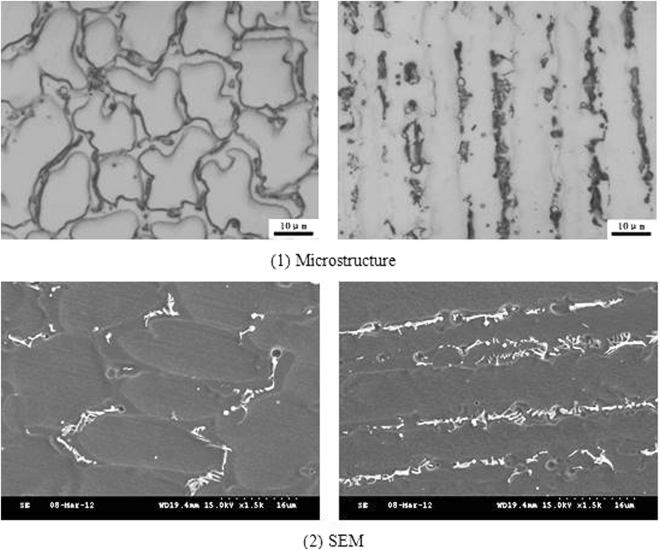
Different cross sections of sub grain boundary in 2# deposited metal.

The microstructure and composition of the 1# and 2# deposited metals were similar to the 3#, where both were austenite and precipitated phases, as presented in Figs 6 and 7. The Creq and Nieq of the 1# deposited metal were 19.1 and 22.9, respectively. The Creq and Nieq of the 2# deposited metal were 19.1 and 23, respectively. The chromium equivalent and nickel equivalent of the two deposited metals were basically the same. Since the copper content of the 2# deposited metal was higher than the copper content of the 1# deposited metal, the nickel equivalent was slightly higher. It was apparent that the chromium and nickel equivalents of the 1# and 2# deposited metals were located at the phase diagram of the austenite region, whereas the solidification was austenitic. The composition analysis was conducted with the micro-area chemical analysis technology in the solidification sub grain boundary and the austenite grains of the metal. The corresponding statistical results are presented in Table 4. Similarly to the 3# deposited metal, the composition of each element in the solidified sub grain boundary was higher compared to the austenite grains.

**Table 4:** Chemical composition of sub-grain boundary and austenite grain in deposited metals (wt.%).

No.	Location	C	Si	Mn	Cr	Ni	Mo	N	Nb	Cu
1#	sub-grain boundary	0.01	0.38	3.61	18.45	16.79	0.99	0.12	0.88	3.81
		8	3	6	7	3	1	6	8	0
	Austenite crystal	0.01	0.29	2.87	17.27	15.90	0.70	0.12	0.45	3.10
		5	7	6	5	3	2	7	7	4
2#	sub-grain boun dary	0.02	0.42	3.76	18.60	17.27	0.94	0.15	0.75	4.63
		3	1	4	9	5	7	2	6	1
	Auste nite crystal	0.02	0.33	2.81	17.4	16.24	0.68	0.14	0.40	3.48
		1	7	9	8	1	3	2	8	2

The contents of niobium in the solidified sub grain boundary and the austenite grains of the three deposited metals were compared. It could be observed that the content of niobium in the solidified sub grain boundary and the austenite grains of the three deposited metals did not differ significantly; the proportion of the niobium in the solidified sub grain boundary was approximately 0.8 wt.%, whereas the proportion of the austenite grains was approximately 0.4 wt.%. Therefore, it could be concluded that the solid solution ratio was the same in all three deposited metals. The Nb, which was not dissolved in the solidified sub grain boundary and the austenite matrix, precipitated out. Due to the high content of insoluble niobium in the 1# and 2# deposited metals, the content of niobium in the non solid solution would consume increased amounts of carbon and nitrogen. The morphology from SEM at different cross sections of the grain boundaries is presented in Figs 6 and 7, where the niobium phase content on the solidified subgrain boundary of the 1# and 2# deposited metal exceeded by far niobium phase content of the 3# deposited metal. From the proportion of the area occupied by the niobium phase in the microstructure of the three deposited metals from SEM, it could be observed that the content of niobium phase in the 1# and 2# deposited metals was approximately 4.5 times the niobium phase content of the 3# deposited metal.

### Solidification grain boundary

**Figure 8:**
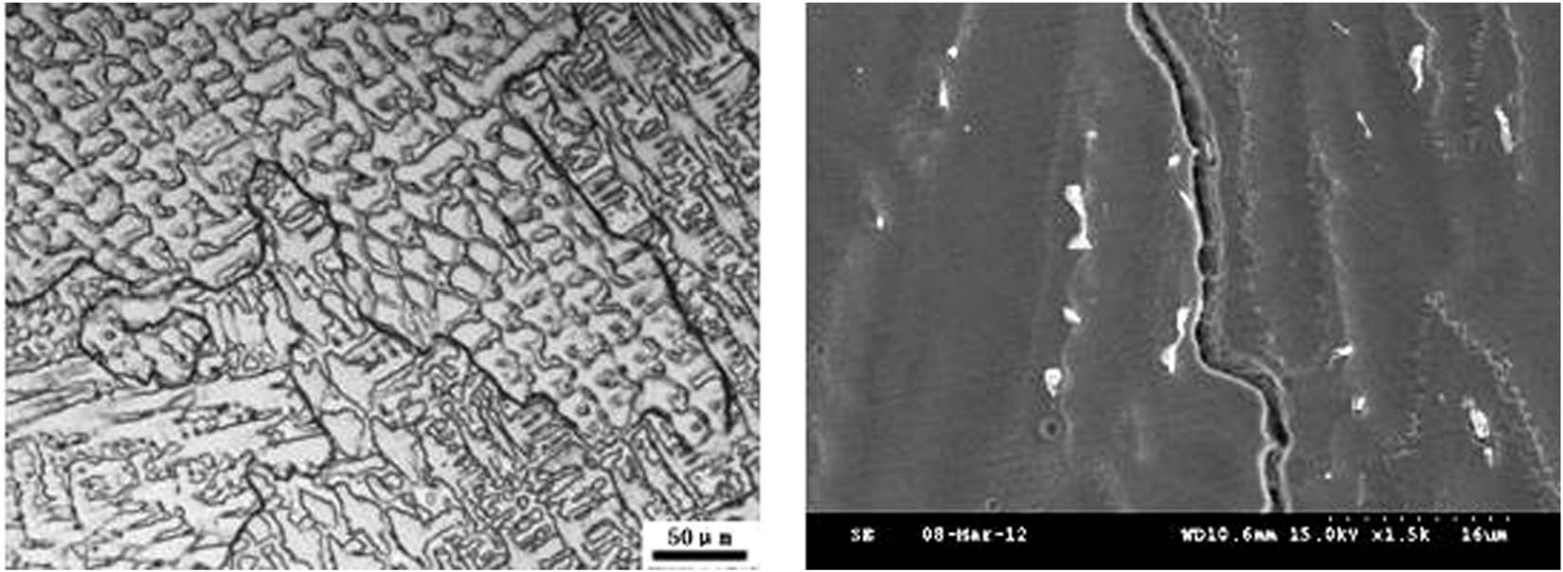
Grain boundaries in 3# deposited metal.

During the weld pool solidification, the crystal grains grew competively along the boundary of the weld pool. The solidified grain boundaries were formed by the intersection of a sub grain beam or a sub grain group and mostly presented in a closed ring distribution, as presented in Fig. 8. Due to the different growth direction and crystal orientation, the solidification grain boundary became the high angle grain boundary, which led to the dislocation network formation along the solidified grain boundaries. Due to the redistribution of the solute during solidification, the alloy elements and impurity elements in the solidified grain boundary were higher. Consequently, it was easy for a low melting point thin film to form at the end of solidification, whereas the low melting point thin film formed solidification cracks. In the SEM morphology, the solidification grain boundaries were a long and narrow gap, as presented in Fig. 8. It was discovered that the solidification grain boundary was quite easily corroded than the solidified sub grain boundaries and the base body. The solidification crack in the austenitic stainless steel weld always formed along the grain boundary.

**Figure 9:**
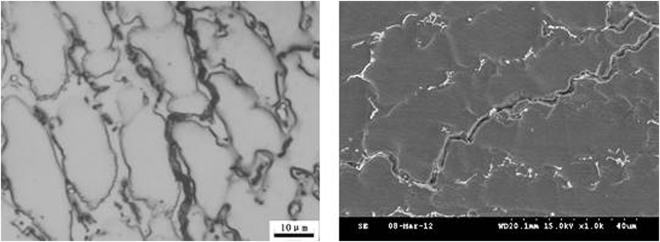
Grain boundaries in 1# deposited metal.

**Figure 10:**
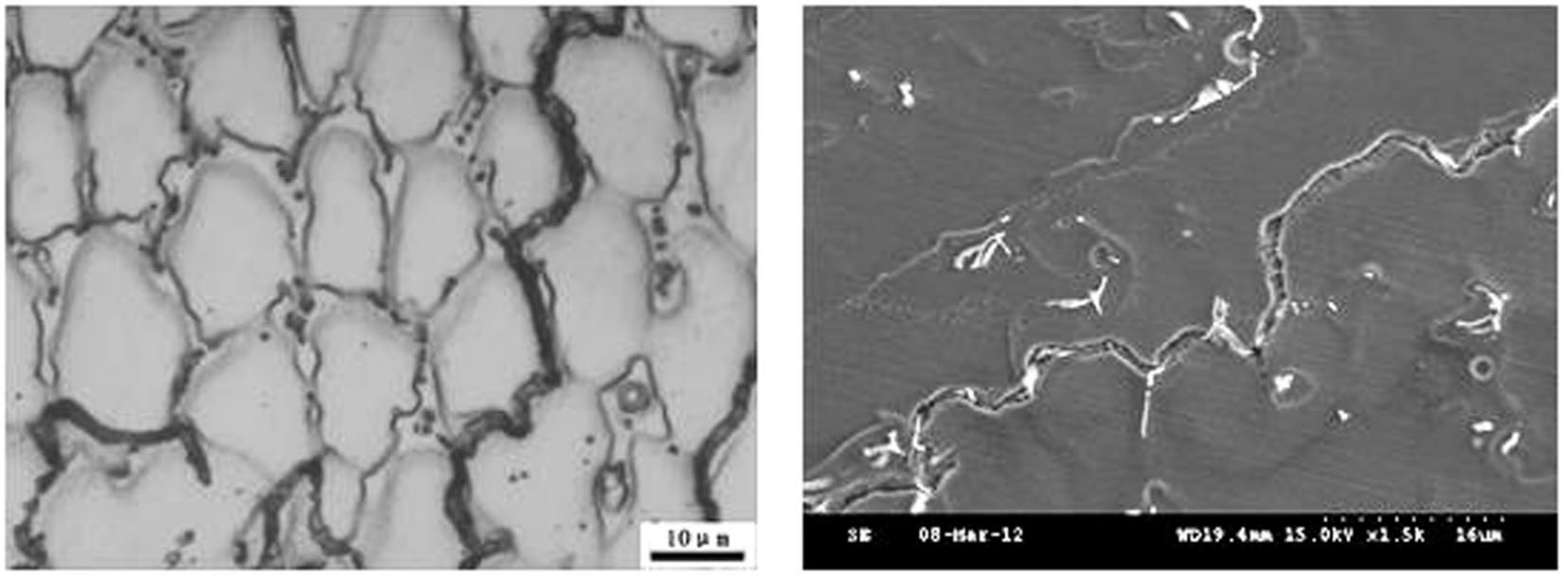
Grain boundaries in 2# deposited metal.

From the SEM morphology of the solidified grain boundaries in Figs 9 and 10, the precipitates were present in the long and narrow gaps of the solidified grain boundaries. Due to the solute redistribution during solidification, the aggregation of niobium, carbon and nitrogen was easy to occur in the solidification grain boundary.

## Effects of Nb and C contents on Nb (C, N) phases

### Calculations of thermodynamic theory

**Figure 11:**
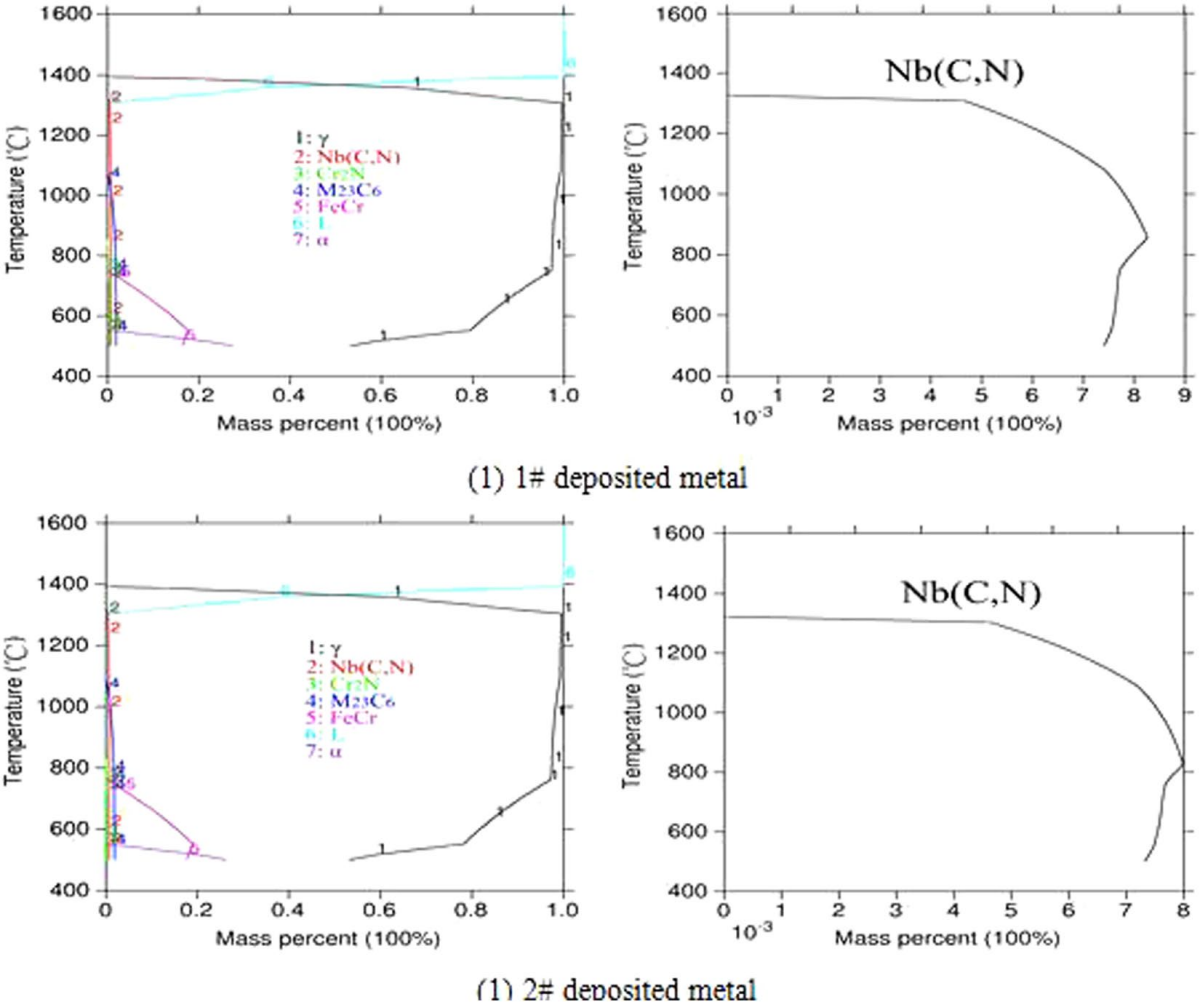
Mass fraction of Thermo-calc precipitates in 1# and 2# deposited metals.

The type and the quantity of the precipitates in the 1# and 2# deposited metal alloys under the equilibrium conditions are presented in Fig. 11, whereas the possible phase of the equilibrium condition were the γ, Nb(C, N), Cr_2_N, M_23_C_6_, FeCr and α. It could be observed that the Nb (C, N) phase precipitation was basically the same in the alloy system of the two deposited metal components, including the temperature of the precipitation, the maximum amount of precipitation and the final precipitation. From the composition of the two alloy systems, the main difference was in the copper content and the Nb (C, N) phase content of the final precipitation approximately 0.73 wt.%. Apparently, under the equilibrium condition, the Nb (C, N) phase precipitation had almost no effect under the equilibrium condition.

**Figure 12:**
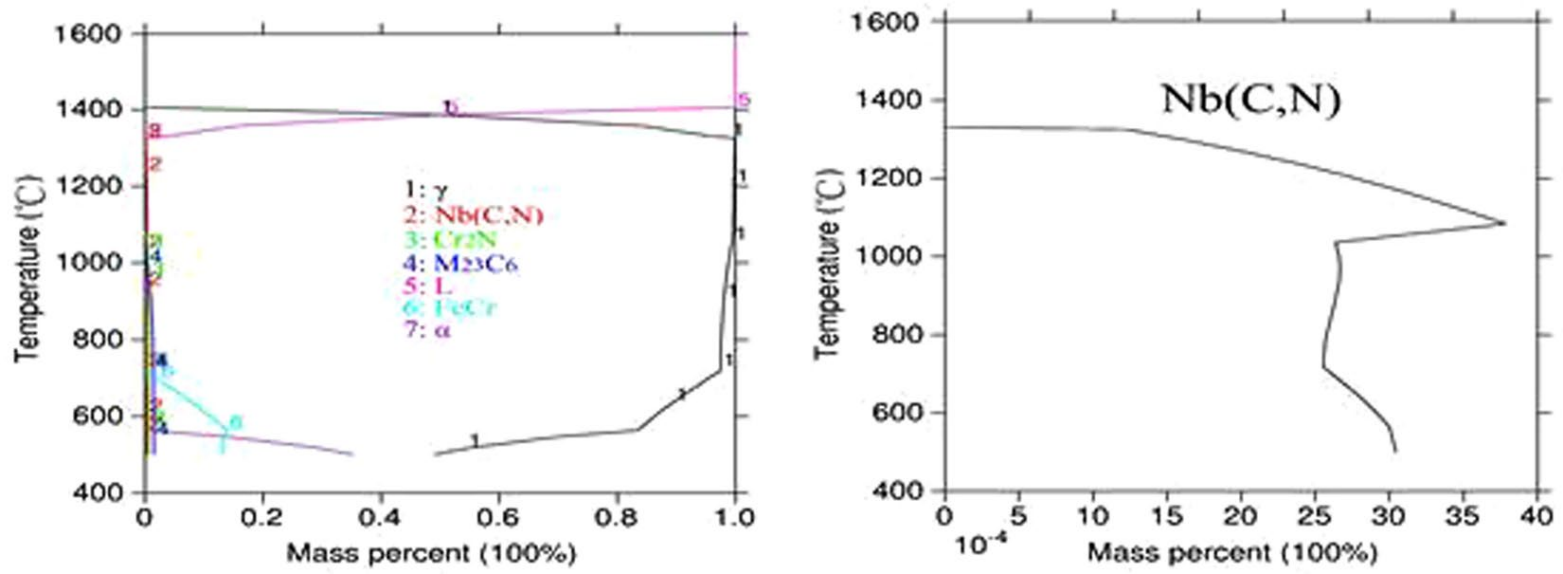
Mass fraction of Thermo-calc precipitates in 3# deposited metal.

According to the calculation results of Thermo-calc, the precipitation phase of the 3# deposited metal alloy system under equilibrium condition was the same as the precipitation phase of the 1# and 2# deposited metal. Compared to the 1# and 2# deposited metal alloys, the contents of carbon and niobium in the 3# deposited metal were lower, as 0.08 wt.% and 0.28 wt.% respectively. From the precipitation curve in Fig. 12, the maximum precipitation temperature of Nb (C, N) was higher than the maximum precipitation temperature of the 1# and 2# deposited metals. It was apparent that the precipitation driving force of the Nb (C, N) phase decreased as the temperature decreased, whereas the contents of carbon and niobium were lower.

### Quantitative phase analysis

**Figure 13:**
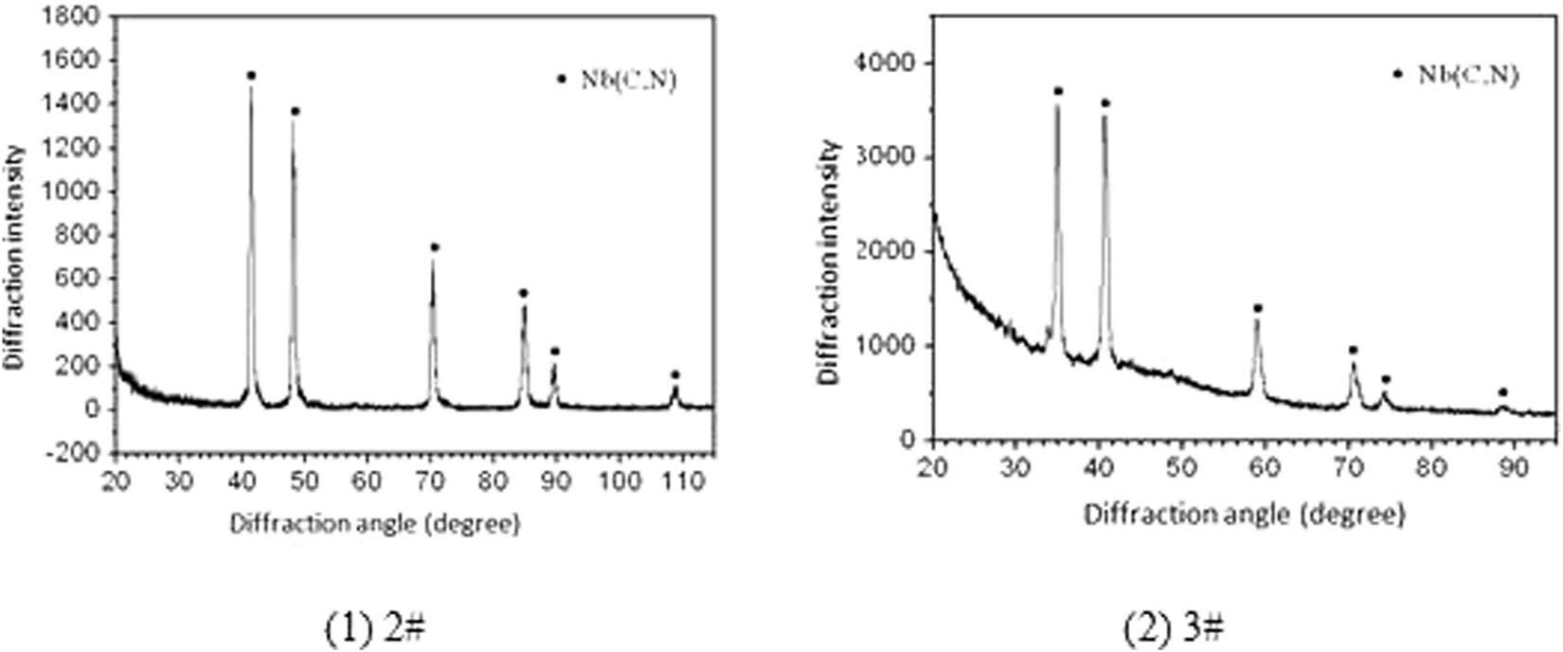
XRD analysis of deposited metals.

The precipitated phases of the 2# and 3# deposited metals were Nb (C, N), as presentedin Fig. 13. The Nb (C, N) is a face centered cubic structure with a lattice constant of 0.439 ~ 0.444 nm. The quantitative phase analysis results demonstrated that the niobium content of the 2# deposited metal was 0.365 wt.% and 0.017 wt.%, whereas the Fe, Cr and Ni were the trace elements. Since molybdenum and niobium could be infinitely soluble, in general, the Nb (C, N) would be the solid solution of a certain amount of molybdenum. The solid solubility of niobium could increase in the substrate by the molybdenum addition. The molybdenum element could increase the solid solubility of niobium in the matrix, consequently the nose temperature of the TTP curve of the precipitation of niobium phase was apparently decreased. The phase analysis results demonstrated that the phases in the deposited metal were only gamma and Nb (C, N), which differed from the species and quantity of the precipitates in the equilibrium condition of the alloy system. This was mainly determined by the non-equilibrium process of the molten pool cool down, whereas it might also be related to the phase analysis accuracy.

The content of copper in austenitic stainless steels is generally in the range of 1 to 4 wt.%, whereas the content of copper in this range has no apparent effect on the steel microstructure. The copper is not the forming element of the niobium phase and it does not occur in the corresponding phases, whereas the effect of copper on the Nb phase precipitation could be neglected. Due to the differences in copper content in the composition of 1# and 2# deposited metals, it was considered that the precipitation of the second phase of the 1# deposited metal was consistent with the precipitation of the second phase of the 2# deposited metal.

The contents of Nb and Mo in the deposited metal were 0.365 wt.% and 0.017 wt.%, respectively, whereas the contents of niobium and molybdenum in the grain boundary and austenite grains were 0.235 wt.% and 0.853 wt.% respectively. According to the calculation results of Thermo-calc, the mass ratio of each element in the Nb (C, N) phases was Nb:N:C = 26:4:1, the nitrogen and carbon contents of niobium phases were 0.056 wt.% and 0.014 wt.%, where the nitrogen and carbon were dissolved into the grain boundary and within the austenite grains were 0.064 wt.% and 0.085 wt.%. For the 3# deposited metal through the same calculation method, the mass ratio of each element in the Nb (C, N) phases was Nb:N:C = 59:11:1, the distribution of niobium, nitrogen, carbon and molybdenum in the 2# and 3# deposited metals are presented in Table 5.

**Table 5:** Distribution of Nb-phase forming elements in the precipitates, grain boundaries and austenite matrix (wt.%).

Distribution of Element	2#				3#			
	Nb	N	C	Mo	Nb	N	C	Mo
Nb phase	0.365	0.056	0.014	0.017	0.167	0.031	0.0028	0.0064
Grain boundary and austenite matrix	0.235	0.064	0.085	0.853	0.113	0.099	0.0772	0.8236
Total	0.60	0.12	0.099	0.87	0.28	0.13	0.08	0.83

It was apparent that the content difference of the Nb(C, N) phases between the 2# and 3# deposited metals was quite high due to the niobium and carbon content differences. The precipitation amount of the 2# deposited metal niobium phase was approximately 2.2 times of the corresponding precipitation amount of 3# deposited metal. Also, the content of niobium in the austenite differed from the content in the austenite grains and the solidified sub-grain boundary. Correspondingly, the nitrogen and carbon contents of the two deposited metals was also different, the nitrogen content of the 3# deposited metal was 0.031 wt.%, whereas the carbon content was only 0.0028 wt.%. The Nb/(C + N) mass ratios of the 2# and 3# deposited metals were 2.73 and 1.33, respectively (the ratio of 2.05). According to the phase analysis results, it was demonstrated that the consumption of niobium in the deposited metal of the 3# and 2# was approximately 60%. This meant that the final consumptions of niobium were 0.365 wt.% and 0.167 wt.% respectively (the ratio of 2.19). It could be observed that the consumption of niobium increased as the Nb/ (C + N) ratio increased and the consumption had an approximately multiple relationship with the ratio of Nb/ (C + N). From the precipitation curve of the Nb (C, N) phase, it could be observed that a certain amount of Nb (C, N) would be produced when the temperature was below 700 °C. During the high temperature extended duration of the deposited metals, the solid solutions of carbon, nitrogen and niobium were precipitated in the form of Nb (C, N), M23C6, or other niobium phases. Therefore, the contents of solid soluble carbon, nitrogen and niobium in the grain boundaries and the matrix would continue to decrease.

## Mechanical properties of deposited metals

**Figure 14:**
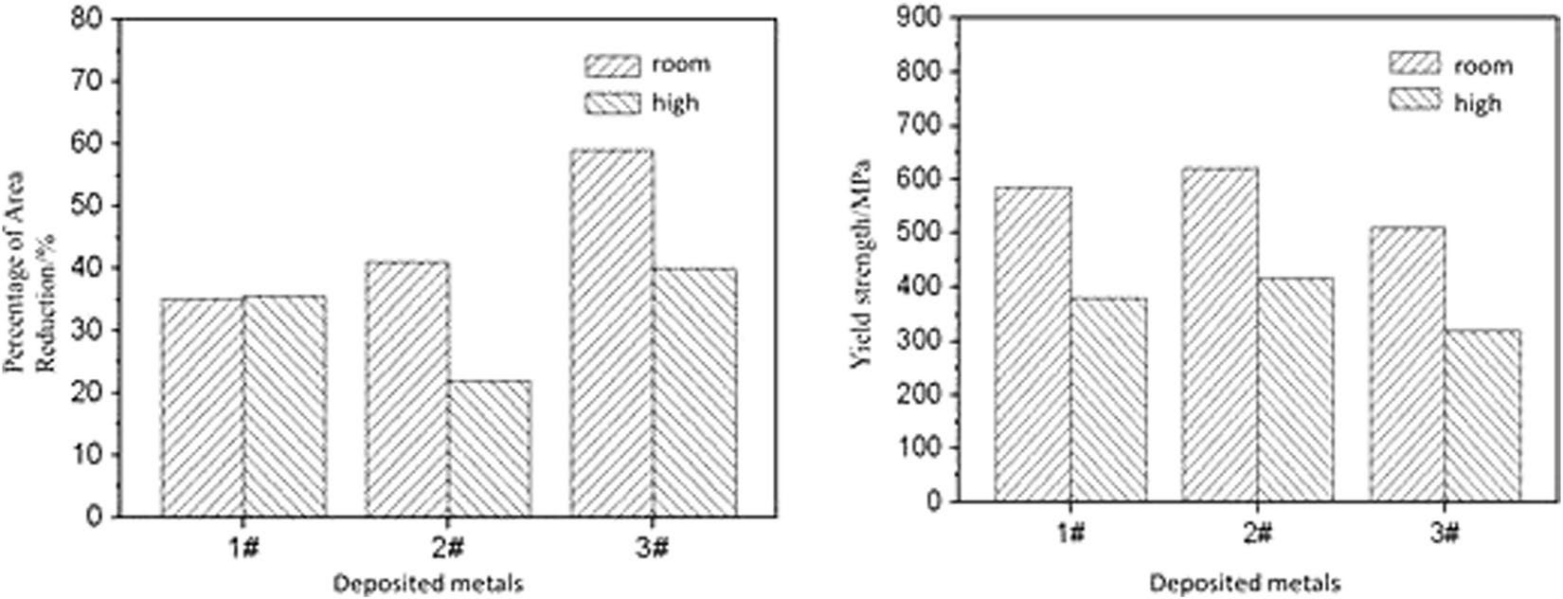
Mechanical properties of deposited metals.

The tensile test results of the three deposited metals at room temperature and the high temperature of 650 °C are presented in Fig. 14. At room temperature, the shrinkage of the 3# deposited metal sample was higher, the plasticity met the requirement and had a high margin. Although the yield strength of the three deposited metals was different, the lower strength of the 3# deposited metal still met the standard requirement. From the impact work, the impact toughness of the 3# deposited metal iswas the highest, whereas the corresponding impact work was 131 J and the impact value of the 1# and 2# deposited metals were 95 J and 109 J respectively. The comprehensive comparison displayed that the room temperature mechanical properties of the 3# deposited metal were better than the mechanical properties of the other two types of deposited metal. From the short-time tensile tests of the three deposited metals at 650 °C, although the performance indexes of the three deposited metals were reduced, the plasticity of the 3# deposited metal was still the best.

The contents of solid solution elements in the deposited metals were added into equation (1), where the following could be available:

1 $$\begin{array}{c}\begin{array}{l}{{\rm{R}}}_{{\rm{p0}}{\rm{.2}}}(1\#)=222.3+7.08{{\rm{d}}}^{-1/2}\\ {{\rm{R}}}_{{\rm{p0}}{\rm{.2}}}(2\#)=222.5+7.08{{\rm{d}}}^{-1/2}\end{array}\\ {{\rm{R}}}_{{\rm{p0}}{\rm{.2}}}(3\#)=212.3+7.08{{\rm{d}}}^{-1/2}\end{array}$$

The chemical composition of the deposited metal from Table 1 emonstrated that the contents of solid solution alloy elements in the 1# and 2# deposited metals were quite low, whereas a slight difference in the copper content existed. Through the data of Table 5 for calculation, the results demonstrated that the solid solution contributions to the yield strength of the alloy was quite low, as 222.3 MPa and 222.5 MPa respectively; also, the solid solution strengthening contribution to the 3# deposited metal was 212.3 MPa. Therefore, the yield strength of the 1#, 2# and 3# deposited metals were 363 MPa, 398 MPa and 298 MPa, respectively.

The microstructure of the deposited metal demonstrated that the yield strength was also determined by the strength of the solidified sub grain boundary and the solidified grain boundary, as well as the distribution of the grain boundary. The tensile test results demonstrated that the yield strength of the 2# deposited metal was 35 MPa, as higher than the yield strength of the 1# deposited metal. Due to the total similar amount of the Nb phase in the 1# and 2# deposited metals, the Nb phase distribution was closely related to the solidification of the sub grain boundaries and the solidified grain boundaries. Therefore, it could be concluded that the niobium phase size, especially the proportion of the high-size Nb phase, as well as the areas of the solidification sub grain boundary and the solidification grain boundary, were the main reasons for the high yield strength of the 2# deposited metal. The yield strength of the 3# deposited metal was the lowest, the quantity and size of the Nb phase were low; therefore the grain boundary area occupied by Nb was relatively low-sized. It could be concluded that the higher yield strength of the 1# and 2# deposited metals mainly originated from the second phase strengthening effect of the Nb phase.

**Figure 15:**
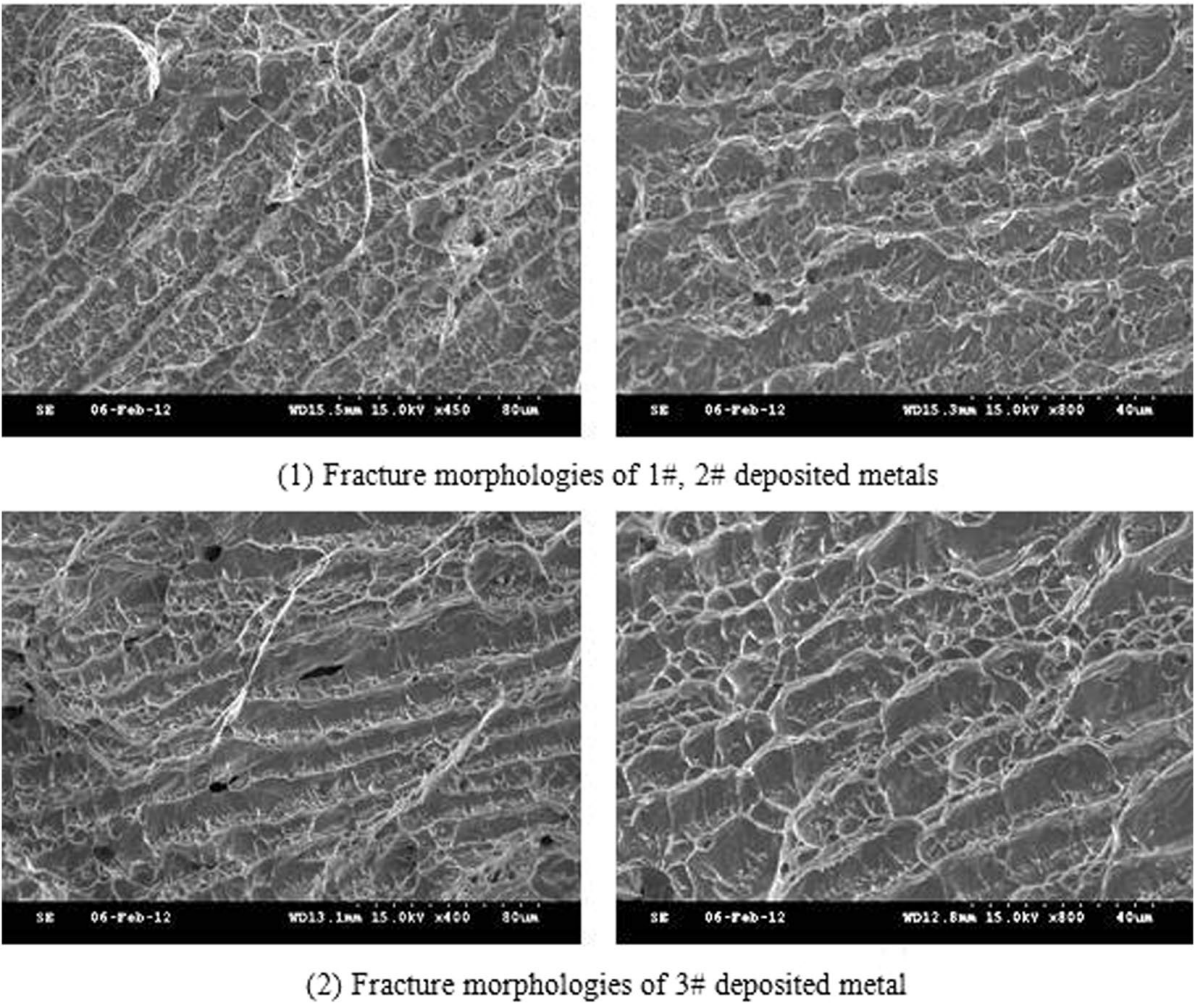
Typical morphology of impact fracture of deposited metals.

The morphology of the impact fracture surface of the three deposited metals was observed, as presented in Fig. 15. Also, the three deposited metals exhibited the typical morphology of the as cast microstructure. It was apparent that the propagation path of cracking continued along the columnar crystal boundary of the deposited metal and the grain boundary was the weak link, which was related to the high amount of the Nb phase on the grain boundary. The fracture morphologies of the three deposited metals were different. The proportion of dimples in the 3# deposited metal was higher. The tear edges were apparently observed at the edge of the dimples. The fracture process displayed good plasticity, which was in agreement with the results of the impact tests.

The niobium phase had high amount and size in the 1# and 2# deposited metals. Due to the corresponding lower toughness compared to the matrix and the grain boundary, the plastic deformation was difficult to accommodate and the plastic work consumed by the microcracks in per unit-area was apparently reduced reduced. Therefore, it was easy to form microcracks with the depolymerization of the grain boundaries and the crack propagation was induced by the growth mechanism of the microporous polymerization. In contrast, the number of niobium phases on the grain boundaries of 3# deposited metal is less, and niobium phases are much smoother than other phases. Therefore, the crack propagation was required to consume higher amount of energy.

## Conclusions

1. The Creq and Nieq composition point of the deposited metals were located in the austenite region and the deposited metals were solidified in the austenitic mode, which was consistent with the prediction of the phase components. The content of each element in the solidified sub-grain boundary was higher than content of each element in the austenite grains.The quantity and size of the Nb-phases in the 3# deposited metal were apparently lower compared to the other two deposited metals. A clear segregation of carbon, niobium and molybdenum existed and the nitrogen accumulation was not apaprent.
2. Differently from the calculation results of Thermo-calc, the phases of the deposited metals were mainly of γ and Nb (C, N). The mean contents of Nb-phases in the deposited metals with 0.6 wt.% and 0.28 wt.% Nb content were approximately 0.452 wt.% and 0.207 wt.%. The relative carbon and nitrogen consumption of the Nb-phases was quite low and most carbon and nitrogen dissolved in the matrix.
3. The content of Nb (C, N) phases in the deposited metal with the 0.6 wt.% Nb content was approximately 2.2 times of the deposited metal with the 0.28 wt.% Nb content. Through the phase analysis quantitative results, the content of niobium in the deposited metal was approximately 60%, and the consumption of Nb also increased as the Nb/ (C + N) ratio increased. The existence ratio of the consumption to the Nb/ (C + N) displayed an approximate multiple relation.
4. The Nb-phases in the deposited metal with the 0.28 wt.% Nb content were lower in amount, weaker in the strengthening effect, lower in yield strength and lower in size than the Nb phases in the deposited metal with the 0.6 wt.% Nb content. The low quantity and low size of Nb phase was conducive to the impact toughness of the deposited metal at room temperature.
